# Applying generalized allometric regressions to predict live body mass of tropical and temperate arthropods

**DOI:** 10.1002/ece3.4702

**Published:** 2018-12-06

**Authors:** Esra H. Sohlström, Lucas Marian, Andrew D. Barnes, Noor F. Haneda, Stefan Scheu, Björn C. Rall, Ulrich Brose, Malte Jochum

**Affiliations:** ^1^ German Centre for Integrative Biodiversity Research (iDiv) Halle‐Jena‐Leipzig Leipzig Germany; ^2^ Institute of Biodiversity Friedrich Schiller University Jena Jena Germany; ^3^ J. F. Blumenbach Institute for Zoology & Anthropology University of Goettingen Goettingen Germany; ^4^ Leipzig University Institute of Biology Leipzig Germany; ^5^ School of Science University of Waikato Hamilton New Zealand; ^6^ Faculty of Forestry, Department of Silviculture Bogor Agricultural University Bogor Indonesia; ^7^ Institute of Plant Sciences University of Bern Bern Switzerland

**Keywords:** allometric scaling, body size, insects, invertebrates, length–mass regression

## Abstract

The ecological implications of body size extend from the biology of individual organisms to ecosystem‐level processes. Measuring body mass for high numbers of invertebrates can be logistically challenging, making length–mass regressions useful for predicting body mass with minimal effort. However, standardized sets of scaling relationships covering a large range in body length, taxonomic groups, and multiple geographical regions are scarce. We collected 6,212 arthropods from 19 higher‐level taxa in both temperate and tropical locations to compile a comprehensive set of linear models relating live body mass to a range of predictor variables. We measured live weight (hereafter, body mass), body length and width of each individual and conducted linear regressions to predict body mass using body length, body width, taxonomic group, and geographic region. Additionally, we quantified prediction discrepancy when using parameters from arthropods of a different geographic region. Incorporating body width into taxon‐ and region‐specific length–mass regressions yielded the highest prediction accuracy for body mass. Using regression parameters from a different geographic region increased prediction discrepancy, causing over‐ or underestimation of body mass depending on geographical origin and whether body width was included. We present a comprehensive range of parameters for predicting arthropod body mass and provide guidance for selecting optimal scaling relationships. Given the importance of body mass for functional invertebrate ecology and the paucity of adequate regressions to predict arthropod body mass from different geographical regions, our study provides a long‐needed resource for quantifying live body mass in invertebrate ecology research.

## 1 INTRODUCTION

Body size is one of the most fundamental traits of living organisms (Peters, 1983). From the individual to the community level, a vast range of properties scale with body size. Body size determines various aspects of an organism's individual biology, such as life history, behavior, range size, movement, and physiology (Bekoff, Diamond, & Mitton, 1981; Hirt, Jetz, Rall, & Brose, 2017; White, Ernest, Kerkhoff, & Enquist, 2007; Woodward et al., 2005). Aspects shaping arthropod communities such as species abundance, biomass production, trophic link structure, and species interaction strengths are also related to the body size of constituent individuals and populations (Belgrano, Allen, Enquist, & Gillooly, 2002; Boudreau, Dickie, & Kerr, 1991; Brose, Williams, & Martinez, 2006; Kalinkat et al., 2013; Rall et al., 2012; Riede et al., 2011). As a result, arthropod body size has substantial impacts on the contribution of individuals and communities to ecosystem processes such as decomposition, pollination or pest control, making it a powerful predictor of ecosystem performance (Barnes et al., 2018).

Most biological rates scale with body size following a power–law relationship (Peters, 1983; White et al., 2007), which has important implications for individual and community ecology. In the early 1930s, Kleiber (1932) proposed an allometric scaling relationship of metabolism with body mass following a ¾ power‐law function, though this has been extensively debated (see Brown, Gillooly, Allen, Savage, & West, 2004; Ehnes, Rall, & Brose, 2011; Kolokotrones, Savage, Deeds, & Fontana, 2010). This power‐law scaling means that smaller animals have a lower per capita metabolic rate than larger ones, though their mass‐specific metabolic rate is higher, yielding distinct patterns of energy demand in populations and communities depending on the relationship between body size and total biomass (Reichle, 1968). Additionally, home‐ and foraging ranges of animals increase with body size, which has been demonstrated for a wide range of organisms, from small invertebrates to large mammals (Greenleaf, Williams, Winfree, & Kremen, 2007; Jetz, Carbone, Fulford, & Brown, 2004; Lindstedt, Miller, & Buskirk, 1986; Swihart, Slade, & Bergstrom, 1988). Due to the allometric scaling of a broad range of physiological and ecological properties, researchers can use general scaling relationships to predict ecological properties from measured values of organism body size (Savage, Deeds, & Fontana, 2008).

While body size is highly useful as a predictive trait for many ecosystem processes, measurement of individual arthropod body masses from community samples is particularly challenging due to their small body size and typically high abundance. As a consequence, researchers might measure only a few individuals of each species and apply an average of these values to all individuals of that species. This practice disregards intraspecific variation that occurs among sampling sites, especially when the sites are distributed along ecological gradients that affect body size (Violle et al., 2012). In order to solve this issue and adequately account for intraspecific variation, the measurement of arthropod body size would have to be simple enough to allow for the processing of high individual numbers. However, in extensive field sampling campaigns, collecting individual body mass data across all samples is often infeasible due to the logistic difficulties of weighing large numbers of individual organisms. Additionally, many ecological disciplines typically require data on live rather than dry body mass to relate body size to a range of ecological attributes. For example, studies investigating arthropod metabolism (Ehnes et al., 2011; Meehan, 2006), interaction strengths and the dimensionality of consumer search space (Pawar, Dell, & Savage, 2012; Vucic‐Pestic, Rall, Kalinkat, & Brose, 2010), movement (Hirt, Lauermann, Brose, Noldus, & Dell, 2017) and size‐abundance relationships (Chown & Steenkamp, 1996; Gouws, Gaston, & Chown, 2011) typically rely on live body mass of organisms. However, dry body mass estimates are more frequently available for arthropods because of the difficulty of accurately measuring their live body mass. This limitation calls for the provision of practical and accurate tools to acquire individual‐level, live arthropod body mass data in order to assess population and community responses in arthropod size structure and investigate corresponding ecosystem processes. Different approaches to indirectly assess body mass have been proposed in the literature. Among others, these include quantitative magnetic resonance (O'Regan, Guglielmo, & Taylor, 2012), clay‐modeling, image analysis or geometric approximation (Llopis‐Belenguer, Blasco‐Costa, & Balbuena, 2018). While these are powerful methods for low sample sizes, obtaining individual body masses for high abundance samples with organisms from many taxonomic groups is infeasible and is where length–mass regressions provide an optimal alternative.

Length–mass regressions have proven to be a powerful tool to predict body mass based on body length measurements (Benke, Huryn, Smock, & Wallace, 1999; Gruner, 2003; Johnston & Cunjak, 1999; Rogers, Buschbom, & Watson, 1977; Schoener, 1980; Wardhaugh, 2013), which are in some cases, easier to obtain than direct measurements of body mass. For living or particularly small organisms, direct measurement of body mass can be difficult and time‐consuming. The length–mass regression approach relies on regression parameters estimated for length–mass relationships. However, finding suitable regression parameters for a given taxon from a specific geographic region is often not possible. This limitation can be problematic because scaling relationships—and thus, their regression parameters—are likely to vary substantially among taxonomic groups and geographic regions; an aspect that has been shown to be especially distinct between tropical and temperate regions (Gruner, 2003; Schoener, 1980; Wardhaugh, 2013). Thus, using length–mass regression parameters from a different geographical region is likely to increase the discrepancy in predictions of body mass. Finally, datasets of length–mass regressions available in the literature are often based on dry body mass measurements. Therefore, researchers requiring live body mass estimates are typically constrained to using rough conversion factors (Peters, 1983) or more elaborate dry mass–fresh mass regressions (e.g., Mercer, Gabriel, Barendse, Marshall, & Chown, 2001), which add further discrepancy to body mass predictions due to the very same sources of variation in length–mass scaling relationships (geographic origin, taxon‐specificity, etc.). Considering the broad application of live body size data in ecological research, there are surprisingly few studies that provide length–body mass regression parameters for terrestrial arthropods, and most studies are restricted to one of either temperate or tropical animals, or to only a few taxonomic groups (Benke et al., 1999; Burgherr & Meyer, 1997; Gruner, 2003; Mercer et al., 2001; Schoener, 1980; Wardhaugh, 2013).

In this paper, we provide an unprecedented dataset of length–mass scaling relationships based on measurements of live body mass and body length of 6,212 terrestrial arthropods from both tropical and temperate geographical regions. We performed length–mass regressions for arthropods, including various combinations of body width, taxonomic group and geographic origin as additional covariables, and compared the accuracy in predicting body mass among these various models. We hypothesized that prediction accuracy improves with an increasing number of additional predictors (e.g., including body width, taxonomic group, and geographic region), as opposed to using only body length as a sole predictor of body mass. Additionally, we expected a higher prediction accuracy when using regression parameters taken from the same geographic region, as opposed to using regression parameters of arthropods from a different geographic region (hereafter, geographically disjunct regression parameters). Our study thus provides a generalized resource for predicting live body mass across an unprecedented range of terrestrial arthropod groups (including 19 orders of Arachnida, Myriapoda, Crustacea, and Insecta), as well as guidance for deciding which scaling relationships to use for predicting arthropod body mass depending on the dataset at hand.

## 2 MATERIALS AND METHODS

### 2.1 Study sites and sampling techniques

To account for different scaling relationships in temperate versus tropical geographical regions, we chose two sampling locations: one temperate location in Germany and one tropical location in Indonesia. Temperate sites were located near Göttingen, Germany (51°32′02″N, 09°56′08″E) at an altitude of around 150 m asl, with a mean annual air temperature of 7.4°C, mean annual precipitation of 700 mm (Heinrichs, Winterhoff, & Schmidt, 2014) and a vegetation growth period from May to September. Tropical sites were located near Jambi City in Sumatra, Indonesia (1°35′24″S 103°36′36″E), at an altitude around 20 m asl. Jambi City has a mean annual air temperature of 25°C and a mean annual precipitation of 2,100 to 2,800 mm (Ishizuka, Tsuruta, & Murdiyarso, 2002). The sampling sites in both regions included wayside vegetation, open grassland areas, and forest strips. Sampling sites were chosen due to their proximity to the laboratory in both regions to ensure a fast and simple workflow, since animals had to be kept alive after collection and living animals could not be stored for more than 8 hr to avoid increased body mass loss.

The temperate organisms were collected in June, July, and August 2014 and the tropical organisms were collected in October, November, and December 2014. Three standard sampling techniques were used in order to cover a broad variety of arthropod taxa and to achieve a sufficient overlap of taxonomic groups from both sampling regions. For active and fast moving ground animals, as well as nocturnal species, live pitfall traps (diameter of 11 cm and height of 12 cm) were used within forest and grassland sites. Pitfall traps were closed with a funnel‐shaped lid to prevent animals from escaping. Pitfall traps were buried so the opening of the pitfall was flush with the surface of the ground. They were installed in the morning and animals were collected after 24 hr to avoid loss of individuals due to predation, drowning, or desiccation. Sweep nets were used in open grassland and wayside vegetation plots to collect animals from within low vegetation, shrubs and small trees to sample stationary, as well as fast‐moving and flying animals. At the forest sites, less mobile animals from within the litter layer were collected via leaf litter sieving. Material from the loose leaf litter (*F*‐Layer) on top of the humus layer was collected and sieved through a coarse‐meshed grid (2 × 2 cm). Animals that fell through the mesh were hand‐collected from a collecting tray and stored in individual vials for further processing.

### 2.2 Morphological measurements and data collection

Arthropods were stored in a refrigerator at 10°C for a maximum of 8 hr after collection to slow down their metabolism and reduce body mass loss. As the goal of our study was to provide length–live body mass regressions, we weighed all arthropods while still alive on a precision scale (to the nearest 0.01 mg) and subsequently stored them in ethanol (75%). For measurements of length and maximum width (to the nearest 0.01 mm), pictures of the dorsal or ventral and lateral view were taken with a Dino‐Lite Digital Microscope (Dino‐Lite Edge; AnMo Electronics Corporation). Afterward, each individual was measured using ImageJ (Version 1.48 k or newer), leaving out appendages to generalize the process. Finally, every individual was identified to family level using “Insects of Australia” (Commonwealth Scientific and Industrial Research Organization (Australia) (1991)), “Spider Families of the World” (Jocqué & Dippenaar‐Schoeman, 2007) and the identification keys of “Brohmer – Fauna von Deutschland”(Schaefer, 2009).

### 2.3 Statistical analysis

All statistical analyses were performed using R Version 3.4.0 (R Core Team, 2015). All larvae and taxa without width measurements were excluded from the main analysis. We present length–mass regressions for these excluded taxonomic groups, along with a range of behavioral, morphological, or taxonomic groups of specific taxa in the Supporting Information (Table S1). Specifically, subgroup regressions are presented for web building and hunting spiders (Araneae), Brachycera, and Nematocera (Diptera), Staphylinidae, beetle larvae and all other beetles aside from larvae and Staphylinidae (Coleoptera), Heteroptera, and all Hemiptera without Heteroptera, larvae of Lepidoptera, Glomerida (only length measurement), Julida (only length measurement) and five morphological subgroups of Hymenoptera.

We log_10_‐transformed body mass, body length, and body width to assure normality of the data and to prevent negative model predictions. Using generalized linear models, we tested the relationship between body mass and length (L), including width (W) and two other covariables. As there was no full‐factorial design for the two factorial independent variables taxonomic group (T) and geographic region (R) (because not all taxa were found in both regions), we created a factorial variable combining these two predictors (“TaxReg”). Note that this implies an interaction between taxonomic group and geographical region that we cannot resolve as long as we use the full dataset. Our most complex model included body length and width (additive, a multiple regression), the factorial variable “TaxReg” and the interactions between each of the two continuous variables and the combined factorial variable (model LWTR). All other models were constructed by reducing the complexity of this overall model by removing independent variables and providing all combinations of these under the above‐described constraints for interactions. Some of the models include taxonomic group (T) or region (R) only or none of the factorial variables, (see Table 1 for the eight models tested and see Supporting Information Methods S1 for a worked example of body mass predictions using each model type). Model fits were compared using the Bayesian information criterion (BIC) and prediction errors obtained through leave‐one‐out cross‐validation (LOOCV) from the R package “boot” (Canty & Ripley, 2017). *R* ^2^ values were calculated using the “rsq”‐function from the R package “rsq”.

**Table 1:** Model comparisons for the eight generalized linear models used to predict live body mass based on different explanatory variables. Models are compared based on BIC, BIC weights , *R* ^2^ and prediction error (mean squared error, *MSE*) of the cross‐validation procedure

Model no.	Model equation	Model parameters	BIC	Δ BIC	BIC weight	*R* ^2^	Prediction error
1 (LWTR)	$\log_{10}\left(\text{mass}\right)∼\text{TaxReg}\;\times {\text{(log}}_{10}{\text{(length) + log}}_{10}\text{(width))}$	Length, width, taxon, region	−7,797.96	0	1	0.972	0.016
2 (LWT)	$\log_{10}\left(\text{mass}\right)∼\text{taxon}\times \left(\log_{10}\left(\text{length}\right)+\log_{10}\left(\text{width}\right)\right)$	Length, width, taxon	−7,601.34	196.62	0	0.970	0.017
3 (LWR)	$\log_{10}\left(\text{mass}\right)∼\text{region}\times \left(\log_{10}\left(\text{length}\right)+\log_{10}\left(\text{width}\right)\right)$	Length, width, region	−4,307.07	3,490.89	0	0.945	0.029
4 (LW)	$\log_{10}\left(\text{mass}\right)∼\log_{10}\left(\text{length}\right)+\log_{10}\left(\text{width}\right)$	Length, width	−4,213.35	3,585.61	0	0.944	0.030
5 (LTR)	$\log_{10}\left(\text{mass}\right)∼\text{TaxReg}\times \log_{10}(\text{length)}$	Length, taxon, region	−1,038.94	6,759.02	0	0.914	0.046
6 (LT)	$\log_{10}\left(\text{mass}\right)∼\text{taxon}\times \log_{10}(\text{length)}$	Length, taxon	−803.49	6,994.47	0	0.910	0.049
7 (LR)	$\log_{10}\left(\text{mass}\right)∼\text{region}\times \log_{10}(\text{length)}$	Length, region	2,909.16	10,707.12	0	0.824	0.093
8 (L)	$\log_{10}\left(\text{mass}\right)∼\log_{10}\left(\text{length}\right)$	Length	3,096.11	10,894.07	0	0.818	0.096

We hypothesized that using regression parameters from different geographic regions likely increases discrepancy in predictions of arthropod body mass. In order to assess this prediction discrepancy, we quantified the proportional difference between predicted and observed body mass using geographically nondisjunct and geographically disjunct regression parameters (i.e., where regression parameters obtained from one geographic region are used to predict body mass of arthropods in a different geographic region) for the two all‐taxa models (models LWR and LR). Specifically, we calculated body mass prediction discrepancy of regression parameters as the log response ratio

$$\Delta =\log_{10}\left(\frac{m_{\mathrm{pred}}}{m_{\mathrm{obs}}}\right),$$

where Δ is the prediction discrepancy, *m_pred_* is the predicted body mass using length–mass regressions and *m_obs_* is observed body mass (obtained by weighing organisms). We then assessed how prediction accuracy varied across the range of body length to ascertain if there might be systematic error in body mass predictions depending on arthropod body size. We applied geographically disjunct and nondisjunct regressions separately to all temperate and all tropical body lengths. Subsequently, we divided predicted by observed body mass values and calculated the decadic logarithm of this ratio. Zero discrepancy thus means that predicted and observed body masses are identical. Positive discrepancy means that predicted body masses are higher than observed body masses and negative discrepancy means that predicted body masses are lower than observed body masses. Given that we use all temperate and tropical body lengths for obtaining the model regressions in the first place and for testing them here, the calculated discrepancy patterns will be symmetrical between temperate and tropical data. For further detail on the calculation and interpretation of prediction accuracy, please refer to Supporting Information Figure S1.

## 3 RESULTS

In total, 6,212 individuals from 19 arthropod taxa were collected, weighed while alive, and measured for body length and width across the tropical and temperate sites. Body length of collected arthropods ranged from 0.62 to 68.12 mm and body mass ranged from 0.01 to 5,108.57 mg (Table 2). As expected, we found a consistent positive scaling relationship for body mass with body length across all collected arthropods (for all arthropod taxa except for temperate Neuroptera in models including body width and body length).

**Table 2:** Taxonomic groups sampled in the two geographic regions (temperate and tropical), including the number of individuals (*n*), number of families, body length range, and body mass range (live body mass) per taxon

Taxonomic group	*n*		No. of families		Length range (mm)		Mass range (mg)	
	Temp.	Trop.	Temp.	Trop.	Temp.	Trop.	Temp.	Trop.
Araneae	519	1,081	16	27	1.01–12.26	0.78–25.71	0.15–212.78	0.01–5,108.57
Coleoptera	382	281	15	21	1.66–35.10	1.10–43.42	0.33–1,067.93	0.05–3,698.96
Dermaptera	60	130	2	3	3.00–13.96	1.87–18.71	2.13–72.06	0.01–92.57
Dictyoptera	—	247	1	6	—	1.69–65.07	—	0.42–1,060.93
Diptera	504	189	31	28	1.49 – 16.82	1.58–23.61	0.07–74.50	0.07–165.17
Geophilomorpha	—	13	—	2	—	7.47–33.54	—	0.29–21.03
Hemiptera	598	454	14	35	1.31–12.05	0.95–23.76	0.27–146.90	0.05–261.53
Hymenoptera	222	371	14	23	1.70–22.26	0.62–31.88	0.06–835.43	0.01–1,664.61
Isopoda	88	88	6	3	2.45–16.16	2.45–16.16	0.81–181.27	0.22–189.52
Lepidoptera	29	87	4	9	3.56–16.23	3.23–27.43	1.67–91.02	0.56–908.65
Lithobiomorpha	161	60	1	1	2.77–23.63	2.22–51.21	0.65–170.65	0.01–439.53
Neuroptera	21	18	2	4	3.79–11.34	3.26–27.29	2.61–17.44	1.33–144.05
Odonata	—	19	—	2	—	23.37–54.83	—	44.96–367.32
Opiliones	89	24	3	3	0.93–7.53	1.09–10.09	0.81–95.02	0.40–165.61
Orthoptera	35	277	2	6	3.79–24.28	1.28–68.12	3.81–417.84	0.14–3,895.10
Polydesmida	12	80	1	1	9.21–19.95	4.02–32.55	9.24–67.25	0.05–205.02
Pseudoscorpionida	—	36	—	2	—	0.95–4.16	1.33–19.91	0.16–2.12
Psocoptera	—	26	—	3	—	1.12–2.92	0.22–0.64	0.11–8.00
Scolopendromorpha	—	11	—	2	—	4.83–41.84	—	0.88–276.18
Total (geogr. region)	2,720	3,492	122	189	0.930–35.1	0.62–68.12	0.06–1,067.93	0.01–5,108.57
Grand total	6,212		243		0.60–68.10		0.01–5,108.57	

**Figure 1:**
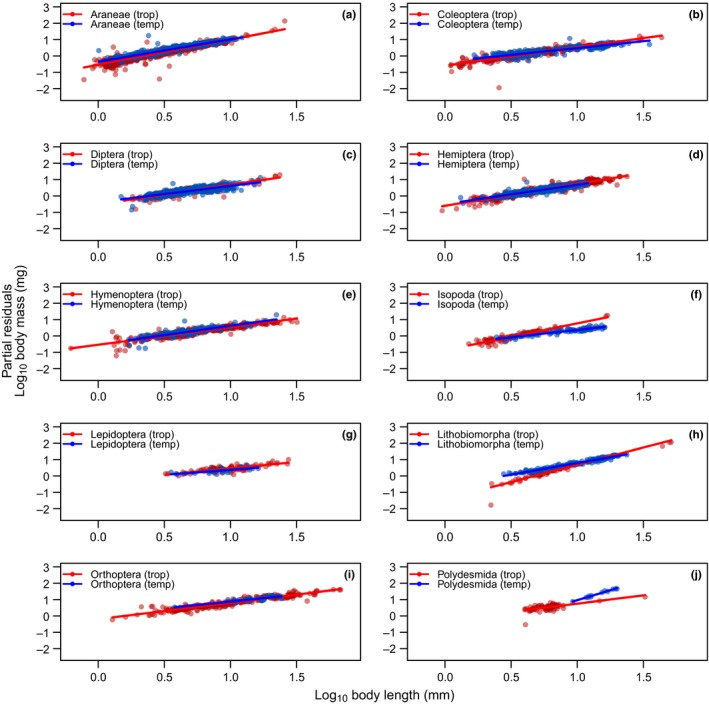
Length–mass regressions of the best fit model, which included body length, maximum body width, taxonomy, and geographic region (LWTR) to predict body mass for the ten most abundant arthropod groups from the temperate (blue) and tropical (red) study areas. The *y*‐axis displays partial residuals and, therefore, shows the effect of body length after correcting for the other variables

The most complex model (Model LWTR, including body length, body width, taxonomic group, and geographic region as predictors) best explained variation in body mass according to BIC selection, *R* ^2^ values and cross‐validation prediction errors (Table 1). We found consistently positive slopes of body mass in relation to body length across our combined factorial variable “TaxReg” including taxonomic groups and regions (Table 3, Figure 1). Thus, the slope of the length–mass relationship varied with body width, taxonomic group and geographic region (e.g., the slope of the length–mass relationship differed between spiders and beetles as well as between temperate and tropical spiders).

**Table 3:** Regression parameters for the eight linear models for live body mass prediction in dependence of body length (L, in mm), maximum body width (W, in mm), taxonomic group (T), and geographic region (R, temperate and tropical). The asterisks indicate significance levels of the regression parameters (***indicates p‐value <0.001; **indicates p‐value <0.01; *indicates p‐value <0.05)

Taxonomic group	Region	Intercept (*a*_x_)	Slope_length_ (*b*_length_)	Slope_width_ (*b*_width_)
Model 1: Length–Width–Taxonomic group–Geographic region–Zone (LWTR)				
Araneae	Temperate	−0.281***	1.368***	1.480***
Coleoptera	Temperate	−0.286***	0.840***	1.954***
Dermaptera	Temperate	−0.369*	1.180***	1.580***
Diptera	Temperate	−0.309***	0.997***	1.595***
Hemiptera	Temperate	−0.420***	1.177***	1.431***
Hymenoptera	Temperate	−0.450***	1.144***	1.724***
Isopoda	Temperate	−0.453**	0.898**	1.756***
Lepidoptera	Temperate	−0.158	0.613***	2.244***
Lithobiomorpha	Temperate	−0.549***	1.416***	1.543***
Neuroptera	Temperate	0.575*	−0.042	2.535***
Opiliones	Temperate	−0.241***	1.353***	1.377***
Orthoptera	Temperate	0.136	0.823**	1.713***
Polydesmida	Temperate	−1.400*	2.443***	0.215
Araneae	Tropical	−0.464***	1.539***	1.448***
Coleoptera	Tropical	−0.523***	1.125***	1.820***
Dermaptera	Tropical	−0.605***	1.301***	1.704***
Dictyoptera	Tropical	−0.326***	0.845***	1.764***
Diptera	Tropical	−0.441***	1.199***	1.399***
Geophilomorpha	Tropical	−0.419	0.964*	1.766***
Hemiptera	Tropical	−0.529***	1.337***	1.260***
Hymenoptera	Tropical	−0.463***	1.070***	1.798***
Isopoda	Tropical	−0.800***	1.646***	1.154***
Lepidoptera	Tropical	−0.256*	0.795***	2.036***
Lithobiomorpha	Tropical	−1.350***	2.112***	0.742
Neuroptera	Tropical	−0.727***	1.506***	1.344***
Odonata	Tropical	−0.513	0.923	1.635
Opiliones	Tropical	−0.384**	2.301***	0.370
Orthoptera	Tropical	−0.117**	1.001***	1.673***
Polydesmida	Tropical	−0.179	1.012***	2.191***
Pseudoscorpionida	Tropical	−0.801***	1.750***	0.300*
Psocoptera	Tropical	−0.936***	2.294***	0.666
Scolopendromorpha	Tropical	−0.962*	1.669***	1.278**
Model 2: Length–Width–Taxonomic group (LWT)				
Araneae	—	−0.410***	1.486***	1.492***
Coleoptera	—	−0.419***	1.001***	1.880***
Dermaptera	—	−0.187**	0.747***	2.228***
Dictyoptera	—	−0.326***	0.845***	1.764***
Diptera	—	−0.375***	1.107***	1.498***
Geophilomorpha	—	−0.419	0.964*	1.766***
Hemiptera	—	−0.472***	1.253***	1.362***
Hymenoptera	—	−0.429***	1.050***	1.801***
Isopoda	—	−0.690***	1.387***	1.393***
Lepidoptera	—	−0.253**	0.785***	2.051***
Lithobiomorpha	—	−0.327**	1.083***	2.058***
Neuroptera	—	−0.515***	1.251***	1.533***
Odonata	—	−0.513	0.923	1.635
Opiliones	—	−0.243***	1.442***	1.262***
Orthoptera	—	−0.095*	0.968***	1.730***
Polydesmida	—	−0.417*	1.245***	1.809***
Pseudoscorpionida	—	−0.801***	1.750***	0.300*
Psocoptera	—	−0.936***	2.294***	0.666
Scolopendromorpha	—	−0.962*	1.669***	1.278 ***
Model 3: Length–Width–Geographic region (LWR)				
—	Temperate	−0.285***	1.040***	1.585***
—	Tropical	−0.371***	1.087***	1.647***
Model 4: Length–Width (LW)				
—	—	−0.340***	1.070***	1.634***
Model 5: Length–Taxonomic group–Geographic region (LTR)				
Araneae	Temperate	−0.733***	2.623***	—
Coleoptera	Temperate	−0.938***	2.501***	—
Dermaptera	Temperate	−0.947***	2.337***	—
Diptera	Temperate	−1.057***	2.489***	—
Hemiptera	Temperate	−0.902***	2.386***	—
Hymenoptera	Temperate	−1.486***	3.018***	—
Isopoda	Temperate	−1.292***	2.950***	—
Lepidoptera	Temperate	−1.274***	2.505***	—
Lithobiomorpha	Temperate	−1.671***	2.780***	—
Neuroptera	Temperate	0.152***	0.888	—
Opiliones	Temperate	−0.364	2.379***	—
Orthoptera	Temperate	−0.640***	2.267***	—
Polydesmida	Temperate	−1.519*	2.595***	—
Araneae	Tropical	−0.862***	2.611***	—
Coleoptera	Tropical	−1.123***	2.616***	—
Dermaptera	Tropical	−1.775***	2.929***	—
Dictyoptera	Tropical	−0.644***	1.913***	—
Diptera	Tropical	−0.973***	2.271***	—
Geophilomorpha	Tropical	−2.917***	2.837***	—
Hemiptera	Tropical	−0.813***	2.189***	—
Hymenoptera	Tropical	−1.422***	2.792***	—
Isopoda	Tropical	−1.268***	2.839***	—
Lepidoptera	Tropical	−1.425***	2.637***	—
Lithobiomorpha	Tropical	−1.884***	2.701***	—
Neuroptera	Tropical	−0.884***	2.112***	—
Odonata	Tropical	−0.499	1.703***	—
Opiliones	Tropical	−0.453***	2.648***	—
Orthoptera	Tropical	−0.775***	2.205***	—
Polydesmida	Tropical	−1.825***	2.726***	—
Pseudoscorpionida	Tropical	−0.942***	2.015***	—
Psocoptera	Tropical	−1.154***	2.710***	—
Scolopendromorpha	Tropical	−2.084***	2.702***	—
Model 6: Length–Taxonomic group (LT)				
Araneae	—	−0.830***	2.637***	—
Coleoptera	—	−1.053***	2.592***	—
Dermaptera	—	−1.316***	2.529***	—
Dictyoptera	—	−0.644***	1.913***	—
Diptera	—	−1.032	2.430***	—
Geophilomorpha	—	−2.917***	2.837***	—
Hemiptera	—	−0.817***	2.237***	—
Hymenoptera	—	−1.401***	2.809***	—
Isopoda	—	−1.322***	2.967***	—
Lepidoptera	—	−1.381***	2.599***	—
Lithobiomorpha	—	−1.888***	2.934***	—
Neuroptera	—	−0.871***	2.010***	—
Odonata	—	−0.499	1.703***	—
Opiliones	—	−0.385***	2.439***	—
Orthoptera	—	−0.791***	2.245***	—
Polydesmida	—	−1.986***	2.944***	—
Psocoptera	—	−1.154***	2.710***	—
Pseudoscorpionida	—	−0.942***	2.015***	—
Scolopendromorpha	—	−2.084***	2.702***	—
Model 7: Length–Geographic region (LR)				
—	Temperate	−0.736***	2.191***	—
—	Tropical	−0.826***	2.159***	—
Model 8: Length (L)				
—	—	−0.792***	2.181***	—

Note

Regression equations for the eight models:

Model 1 (LWTR): log_10_ (body mass) = *a*_taxon region_ + *b*_length_ _taxon region_ × log_10_ (body length) + *b*_width taxon region_ × log_10_(body width).

Model 2 (LWT): log_10_ (body mass) = *a*_taxon_ + *b*_length_ _taxon_ × log_10_ (body length_taxon_) + *b*_width taxon_ × log_10_ (body width).

Model 3 (LWR): log_10_ (body mass) = *a*_region_ + *b*_length_ _region_ × log_10_ (body length) + *b*_width region_ × log_10_ (body width).

Model 4 (LW): log_10_ (body mass) = *a* + *b*_length_ × log_10_ (body length) + *b*_width_ × log_10_ (body width).

Model 5 (LTR): log_10_ (body mass) = *a*_taxon region_ + *b*_taxon_ _region_ × log_10_ (body length).

Model 6 (LT): log_10_ (body mass) = *a*_taxon_ + *b*_taxon_ × log_10_ (body length).

Model 7 (LR): log_10_ (body mass) = *a*_region_+ *b*_region_ × log_10_ (body length).

Model 8 (L): log_10_ (body mass) = *a* + *b* × log_10_ (body length).

The eight different models explained between 81.8% (model L, least complex model) and 97.2% (model LWTR, most complex model) of the total variance in body mass (Table 1). According to BIC, *R* ^2^ and the cross‐validation comparisons, the four models that included body width as a covariate explained more variation in body mass than models that only included body length as a predictor (Table 1).

**Figure 2:**
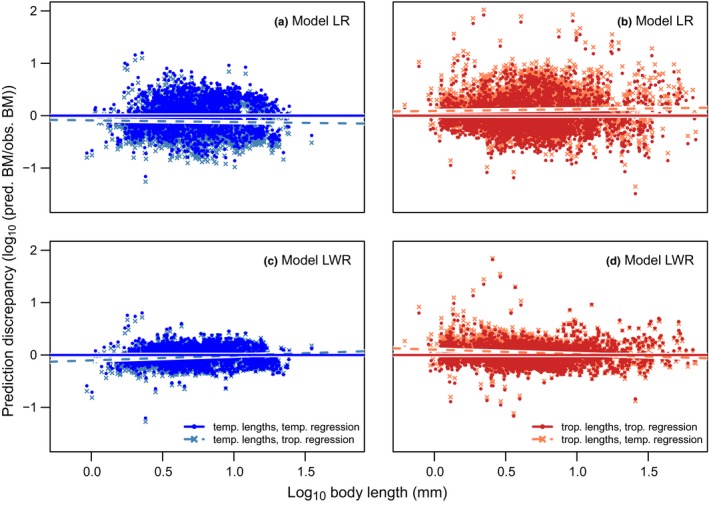
Prediction discrepancy (log response ratio of predicted vs. observed body mass values) for temperate (blue datapoints, panels a and c) and tropical (red datapoints, panels b and d) arthropod body mass obtained by using geographically disjunct (light‐blue crosses and light red crosses) and nondisjunct (dark‐blue points and dark‐red points) regression parameters for the LR (a and b) and LWR (c and d) models. LR = length ***×*** region and LWR = (length + width) ***×*** region models. The lines show the linear model of the log response ratio of predicted and observed body mass values and body length by using geographically disjunct (dashed lines) and geographically nondisjunct lines (solid lines). For further explanation of the presented patterns, please refer to Supporting Information Figure S1

Finally, to test if the application of geographically disjunct regression parameters increases discrepancy in body mass predictions, we calculated body mass using geographically disjunct and geographically nondisjunct regression parameters and quantified the difference to observed body mass. When quantifying the difference between body mass estimates from geographically nondisjunct and disjunct regression parameters, we found that the application of geographically disjunct parameters for whole‐fauna regressions led to increased prediction discrepancy of body mass when compared to using nondisjunct regression parameters (Figure 2). Whether this prediction discrepancy leads to an under‐ or overestimation of body mass depended on the geographic region and the morphological traits used to predict body mass. With body length as the only morphological predictor (Model LR), body mass of temperate arthropods was underestimated on average by 23% (geometric‐mean ratio = 0.77) using tropical regression parameters (Figure 2a), whereas tropical arthropod body mass was overestimated on average by 29% (geometric‐mean ratio = 1.29) when using temperate regression parameters (Figure 2b). Interestingly, when using model LR, prediction discrepancy increased with increasing body length for both temperate and tropical arthropods using geographically disjunct regression parameters (Figure 2a,b). In contrast, when body width was included in the model, the geographically disjunct regression prediction discrepancy shifted between overestimation and underestimation with increasing body length. For temperate arthropods, geographically disjunct models tended to underestimate predicted body mass at small body lengths and overestimate predicted body mass at large body lengths, with an average underestimation of 8% (geometric‐mean ratio = 0.92) (Figure 2c). In contrast, body mass of tropical arthropods was overestimated at smaller body lengths and underestimated at larger body lengths when using geographically disjunct regression parameters in model LWR, with an average overestimation of 11% (geometric‐mean ratio = 1.11) (Figure 2d). For further background on the prediction accuracy methodology and results, please refer to the Supporting Information).

## 4 DISCUSSION

A wide range of individual‐ to community‐level characteristics are influenced by body size, including abundance, metabolic rate, movement speed, or growth rate (Gillooly, Brown, West, Savage, & Charnov, 2001; Hirt, Jetz, et al., 2017; White et al., 2007). In order to make realistic predictions of these measures, it is essential to have reliable body mass data of target organisms. In our dataset consisting of 6,212 organisms spanning 19 taxa from both tropical and temperate geographic regions, we found an overall positive power‐law relationship between body mass and body length across taxonomic groups and the tropical and temperate geographic regions. The only exception to this universal trend was for temperate Neuroptera, which showed a negative relationship between body mass and body length in models that also included body width. A decoupling of length and width through a combination of morphologically distinct individuals and low replication in this group likely caused an average increase in body length without a proportional average increase in body width, resulting in long but thin organisms.

Generally, adding body width as an additional morphological predictor strongly improved body mass prediction accuracy. This increase in model performance is probably due to certain groups where the body length‐to‐width ratio is considerably different to the average of all taxonomic groups (e.g., Staphylinid beetles have a higher body length‐to‐width ratio than other beetle families). Thus, using body length as the only predictor of body mass at the order level is almost certainly insufficient to capture the morphological variation present within taxonomic groups. Therefore, we expected that the incorporation of body width as an additional predictor in our models should increase the accuracy of body mass predictions at the order level. Consistent with our expectations, we found that including body width into the estimation of body mass resulted in a strong improvement of prediction accuracy, in comparison to using body length, alone, as a single predictor of body mass. Moreover, incorporating only body width as an additional predictor yielded higher prediction accuracy than incorporating taxonomic group and geographic region into the models. Body mass is related to the volume of an organism, which can be described by length, width and height. Hence, adding height to predict body mass could lead to more accurate body mass estimations than using only body length and width. Measuring another morphological trait of an organism, however, increases the time needed for processing samples, presenting a trade‐off between maximizing prediction accuracy and minimizing time spent measuring traits. As more than 97% of the variance in body mass was described by length, width, taxonomic group, and geographic region, the benefit of adding body height would unlikely outweigh the added workload. Indeed, previous studies have shown that including body shape (i.e., body length and width) instead of taxonomy lead to more accurate body mass estimates at the order level, but not at higher taxonomic resolution (Gruner, 2003; Wardhaugh, 2013). Our results strongly support the finding that the accuracy in predicting body mass improves with adding further morphological traits, which are related to volume, in addition to body length for scaling relationships conducted at the order level.

Besides body width, taxonomic group and geographic origin of the arthropods also influenced the relationship between body length and body mass. This interaction is likely because variation in arthropod body size is influenced by a range of other factors such as evolutionary history and environmental variation (Chown & Gaston, 2010). For example, Bergmann's rule proposes that body size increases with latitude, though the opposite has been observed for arthropods (Mousseau, 1997). In general, these concepts suggest that the body size of arthropods depends strongly on their geographic origin, particularly with respect to latitude. Therefore, we expected that the application of geographically disjunct regression parameters from tropical and temperate regions could lead to significant prediction discrepancy in arthropod body mass. If researchers are unable to use regression parameters from data collected in a similar geographic region to their study site (due to a lack of available scaling relationships), this could have important consequences for the body mass‐related results drawn from their studies. Consistent with our expectations, we found that the use of geographically disjunct length–mass regression parameters led to inaccurate body mass predictions ranging between average prediction discrepancies of 8% to 23%, depending on the model used. The patterns presented in Figure 2 are caused by the underlying differences between the temperate and tropical length–mass relationships of our study (Supporting Information Figure S1). For a given body length, temperate arthropods had on average higher body mass than tropical arthropods in our dataset. The difference between the relationships increased with body length (see Supporting Information Figure S1 for further explanation). Consequently, when only body length was used as a morphological predictor, body mass prediction discrepancy of geographically disjunct regressions increased with increasing body length of arthropods. This has important consequences for the quality of body mass data, as our results suggest that body mass of longer arthropods will be more severely over‐ or underestimated than that of shorter arthropods. Therefore, our results highlight a potential systematic bias of decreasing prediction accuracy with increasing body length when applying regression parameters from different geographical regions. Ultimately, studies investigating body size responses to environmental conditions and the resulting impacts on ecosystem functioning rely on accurate calculations of body mass. Therefore, it is essential for such studies to use length–mass regression parameters that are obtained from similar geographic origins as the organisms for which body mass is being predicted.

In addition to the potential prediction discrepancy caused by using geographically disjunct regression parameters, using length–mass regressions obtained from organisms collected in a different season might reduce the accuracy of body mass estimations. It has been demonstrated, that body size can vary across seasons as a consequence of temperature variation (Horne, Hirst, & Atkinson, 2017). Hence, our temperate length–mass regressions will likely be most accurate when used for organisms collected during the main vegetation growth period. Furthermore, some animals collected from pitfall traps may have been captured directly after the traps were set and could, therefore, have either starved for up to 32 hr or larger predators could potentially have fed on smaller organisms and temporarily increased their body mass. However, only 421 organisms were captured using pitfall traps, while the majority of arthropods (5,700 organisms) were captured using litter sieving and sweep nets. Moreover, given that studies typically require body mass data for temperate arthropods during their active period and that the only exception to the general positive power‐law relationships was found for a low replication taxon in one geographical region, our dataset of length–mass regressions still provides a useful and robust tool to estimate arthropod body mass.

Our study provides a highly comprehensive set of regression parameters for predicting live body mass of terrestrial arthropods. This set of regression parameters is useful for researchers wishing to quantify body mass of arthropods across a range of underlying morphological traits, taxonomic identities, and different geographical regions. By incorporating all combinations of geographic region, taxonomic group and body width in our allometric models, our results allow investigators to choose length–mass regression parameters for predicting body mass across a broad variety of arthropod datasets. Additionally, we provide an explicit estimation of the prediction discrepancy caused by using geographically disjunct regression parameters, to assist in deciding which regression parameters will be the most appropriate for predicting arthropod body mass for a given dataset. In summary, our results will aid future studies in accurately assessing body mass of arthropods, thus increasing our ability to further explore the ecological implications of body size.

## CONFLICT OF INTERESTS

The authors declare no competing interests.

## AUTHOR CONTRIBUTIONS

LM, ADB, UB, and MJ conceived and designed the study, EHS, LM and MJ carried out the field and laboratory work, EHS and BCR analyzed the data, and all authors interpreted the results. EHS and LM wrote the first draft and all authors contributed substantially to the writing.

## DATA ACCESSIBILITY

All underlying data are available from the Dryad Digital Repository https://doi.org/10.5061/dryad.vk24fr1.
