# Supplementary material for: Applying generalized allometric regressions to predict live body mass of tropical and temperate arthropods
Source: Ecol Evol. 2018 Dec 6;8(24):12737–49. doi: 10.1002/ece3.4702 (PMC6308897; doi:10.1002/ece3.4702)
Supplement: Supplementary file 1 [file ECE3-8-12737-s001.docx]

**Table S1**: Regression parameters for four linear models for live body mass prediction in dependence of body length (L, in mm), maximum body width (W, in mm), group (T) (classified by taxonomy, behaviour or morphology) and geographic region (R, temperate and tropical).

| Group | Region | Intercept (a_x_) | Slope_length_  (b_length_) | Slope_width_  (b_width_) |
| --- | --- | --- | --- | --- |
| **Model 1: Length-Width-Group-Geographic region-Zone (LWTR)** | | | | |
| Araneae  (webbuilding) | temperate | -0.340 | 1.502 | 1.358 |
| Araneae  (hunting) | temperate | -0.133 | 1.077 | 1.663 |
| Coleoptera  (larvae) | temperate | -0.312 | 0.893 | 2.054 |
| Coleoptera  (other) | temperate | -0.438 | 1.290 | 1.451 |
| Coleoptera  (Staphylinidae) | temperate | -0.554 | 1.078 | 1.792 |
| Diptera  (Brachycera) | temperate | -0.232 | 0.865 | 1.678 |
| Diptera  (Nematocera) | temperate | -0.736 | 1.596 | 1.352 |
| Hemiptera  (Heteroptera) | temperate | -0.431 | 1.187 | 1.424 |
| Hemiptera  (other) | temperate | -0.339 | 0.990 | 0.823 |
| Hemiptera  (juvenile) | temperate | -0.673 | 1.949 | 1.710 |
| Hymenoptera 1 | temperate | -0.618 | 1.367 | 1.794 |
| Hymenoptera 2 | temperate | -0.230 | 0.759 | 2.002 |
| Hymenoptera 3 | temperate | 0.090 | 0.244 | 2.824 |
| Hymenoptera 4 | temperate | -0.500 | 1.260 | 1.613 |
| Hymenoptera 5 | temperate | -0.336 | 1.013 | 1.927 |
| Araneae  (webbuilding) | tropical | -0.446 | 1.511 | 1.288 |
| Araneae  (hunting) | tropical | -0.445 | 1.477 | 1.580 |
| Coleoptera  (larvae) | tropical | -0.632 | 1.346 | 1.824 |
| Coleoptera  (other) | tropical | -0.490 | 1.113 | 1.812 |
| Coleoptera  (Staphylinidae) | tropical | -0.720 | 1.175 | 0.811 |
| Dictyoptera  (Blattodea) | tropical | -0.805 | 1.889 | 0.830 |
| Dictyoptera  (Isoptera) | tropical | -0.588 | 1.544 | 1.094 |
| Dictyoptera  (Mantodea) | tropical | -0.336 | 0.522 | 2.590 |
| Diptera  (Brachycera) | tropical | -0.451 | 1.242 | 1.317 |
| Diptera  (Nematocera) | tropical | -0.471 | 1.234 | 1.438 |
| Hemiptera  (Heteroptera) | tropical | -0.555 | 1.353 | 1.232 |
| Hemiptera  (other) | tropical | -0.507 | 1.311 | 1.340 |
| Hymenoptera 1 | tropical | -0.548 | 1.166 | 1.759 |
| Hymenoptera 2 | tropical | -0.317 | 0.951 | 1.759 |
| Hymenoptera 3 | tropical | -0.528 | 1.378 | 1.431 |
| Hymenoptera 4 | tropical | 0.022 | 0.203 | 3.373 |
| Hymenoptera 5 | tropical | -0.281 | 0.853 | 1.854 |
| Lepidoptera  (larvae) | tropical | -0.277 | 0.984 | 2.024 |
| **Model 2: Length-Width-Group (LWT)** | | | | |
| Araneae  (webbuilding) | - | -0.406 | 1.561 | 1.315 |
| Araneae  (hunting) | - | -0.400 | 1.398 | 1.677 |
| Coleoptera  (larvae) | - | -0.537 | 1.185 | 1.874 |
| Coleoptera  (other) | - | -0.472 | 1.244 | 1.575 |
| Coleoptera  (Staphylinidae) | - | -0.768 | 1.406 | 1.312 |
| Diptera  (Brachycera) | - | -0.338 | 1.051 | 1.517 |
| Diptera  (Nematocera) | - | -0.623 | 1.446 | 1.310 |
| Hemiptera  (Heteroptera) | - | -0.486 | 1.262 | 1.354 |
| Hemiptera  (other) | - | -0.460 | 1.237 | 1.420 |
| Hemiptera  (juvenile) | - | -0.673 | 1.949 | 0.823 |
| Hymenoptera 1 | - | -0.493 | 1.105 | 1.817 |
| Hymenoptera 2 | - | -0.279 | 0.886 | 1.827 |
| Hymenoptera 3 | - | 0.320 | 0.944 | 1.953 |
| Hymenoptera 4 | - | -0.400 | 1.084 | 1.815 |
| Hymenoptera 5 | - | -0.244 | 0.821 | 1.963 |
| **Model 5: Length-Group-Geographic region (LTR)** | | | | |
| Araneae  (webbuilding) | temperate | -0.777 | 2.693 | - |
| Araneae  (hunting) | temperate | -0.501 | 2.256 | - |
| Coleoptera  (larvae) | temperate | -1.241 | 2.220 | - |
| Coleoptera  (other) | temperate | -0.915 | 2.581 | - |
| Coleoptera  (Staphylinidae) | temperate | -1.578 | 2.635 | - |
| Diptera  (Brachycera) | temperate | -1.052 | 2.499 | - |
| Diptera  (Nematocera) | temperate | -1.215 | 2.350 | - |
| Glomerida | temperate | -0.747 | 2.510 | - |
| Hemiptera  (Heteroptera) | temperate | -0.906 | 2.373 | - |
| Hemiptera  (other) | temperate | -0.977 | 2.650 | - |
| Hemiptera  (juvenile) | temperate | -1.056 | 2.932 | - |
| Hymenoptera 1 | temperate | -1.309 | 2.744 | - |
| Hymenoptera 2 | temperate | -1.117 | 2.730 | - |
| Hymenoptera 3 | temperate | -1.530 | 2.929 | - |
| Hymenoptera 4 | temperate | -1.197 | 2.379 | - |
| Hymenoptera 5 | temperate | -1.345 | 2.771 | - |
| Julida | temperate | -1.784 | 2.591 | - |
| Araneae  (webbuilding) | tropical | -0.774 | 2.447 | - |
| Araneae  (hunting) | tropical | -0.894 | 2.663 | - |
| Coleoptera  (larvae) | tropical | -1.234 | 2.316 | - |
| Coleoptera  (other) | tropical | -0.928 | 2.552 | - |
| Coleoptera  (Staphylinidae) | tropical | -0.882 | 1.322 | - |
| Dictyoptera  (Blattodea) | tropical | -1.085 | 2.658 | - |
| Dictyoptera  (Isoptera) | tropical | -1.136 | 2.681 | - |
| Dictyoptera  (Mantodea) | tropical | -0.992 | 1.946 | - |
| Diptera  (Brachycera) | tropical | -0.892 | 2.289 | - |
| Diptera  (Nematocera) | tropical | -1.279 | 2.335 | - |
| Hemiptera  (Heteroptera) | tropical | -0.839 | 2.182 | - |
| Hemiptera  (other) | tropical | 0.812 | 2.238 | - |
| Hymenoptera 1 | tropical | -1.446 | 2.747 | - |
| Hymenoptera 2 | tropical | -1.152 | 2.650 | - |
| Hymenoptera 3 | tropical | -1.322 | 2.916 | - |
| Hymenoptera 4 | tropical | -1.392 | 2.579 | - |
| Hymenoptera 5 | tropical | -1.093 | 2.163 | - |
| Julida | tropical | -1.368 | 2.116 | - |
| Lepidoptera  (larvae) | tropical | -1.809 | 2.800 | - |
| **Model 6: Length-Group (LT)** | | | | |
| Araneae  (webbuilding) | - | -0.786 | 2.626 | - |
| Araneae  (hunting) | - | -0.869 | 2.663 | - |
| Coleoptera  (larvae) | - | -1.229 | 2.244 | - |
| Coleoptera  (other) | - | -0.927 | 2.581 | - |
| Coleoptera  (Staphylinidae) | - | -1.352 | 2.326 | - |
| Diptera  (Brachycera) | - | -0.997 | 2.427 | - |
| Diptera  (Nematocera) | - | -1.265 | 2.349 | - |
| Hemiptera  (Heteroptera) | - | -0.819 | 2.223 | - |
| Hemiptera  (other) | - | -0.859 | 2.313 | - |
| Hymenoptera 1 | - | -1.380 | 2.712 | - |
| Hymenoptera 2 | - | -1.080 | 2.616 | - |
| Hymenoptera 3 | - | -1.454 | 3.069 | - |
| Hymenoptera 4 |  | -1.207 | 2.387 | - |
| Hymenoptera 5 | - | -1.127 | 2.298 | - |
| Julida | - | -1.751 | 2.562 | - |

Regression equations for the eight models:

Model 1 (LWTR): log_10_(body mass) = a_taxon region_ + b_length taxon region_ × log_10_(body length) + b_width taxon region_ × log_10_(body width)

Model 2 (LWT): log_10_(body mass) = a_taxon_ + b_length taxon_ × log_10_(body length) + b_width taxon_ × log_10_(body width)

Model 5 (LTR): log_10_(body mass) = a_taxon region_ + b_taxon region_ × log_10_(body length)

Model 6 (LT): log_10_(body mass) = a_taxon_ + b_taxon_ × log_10_(body length)

Hymenoptera 1 includes the following families Formicidae, Dryinidae, Muttilidae, Embolemidae

Hymenoptera 2 includes the following families: Apidae, Colletidae, Megachilidae, Pamphilidae, Scoliidae, Sphecidae, Tenthredinidae, Vespidae

Hymenoptera 3 includes the following families: Ceraphronidae, Chalcididae, Diapriidae, Eulophidae, Eupelmidae, Eurytomidae, Evaniidae, Perilampidae, Proctotrupidae, Proctotrupidae, Scelionidae

Hymenoptera 4 includes the family Ichneumonidae

Hymenoptera 5 includes the following families: Bethylidae, Braconidae, Gasteruptiidae, Tiphiidae

**Prediction discrepancy – methodology and patterns in presented results**

We calculated body mass prediction discrepancy of using geographically disjunct and non-disjunct regression parameters for all temperate and tropical body lengths from our study using two different models. This procedure resulted in the eight lines (temperate and tropical body lengths, temperate and tropical regressions, 2 models) presented in Fig. 2. Thus, for example the prediction discrepancy for temperate body masses using temperate body lengths with tropical regression parameters (geographically disjunct) is presented by the dashed lines in Fig. 2a and c. We calculated prediction discrepancy as:

$\Delta={log}_{10}\left( \frac{m_{pred}}{m_{obs}} \right)$,

where Δ is the prediction discrepancy of body mass, *m_pred_* is the predicted body mass using length-mass regressions and *m_obs_* is observed body mass.

*
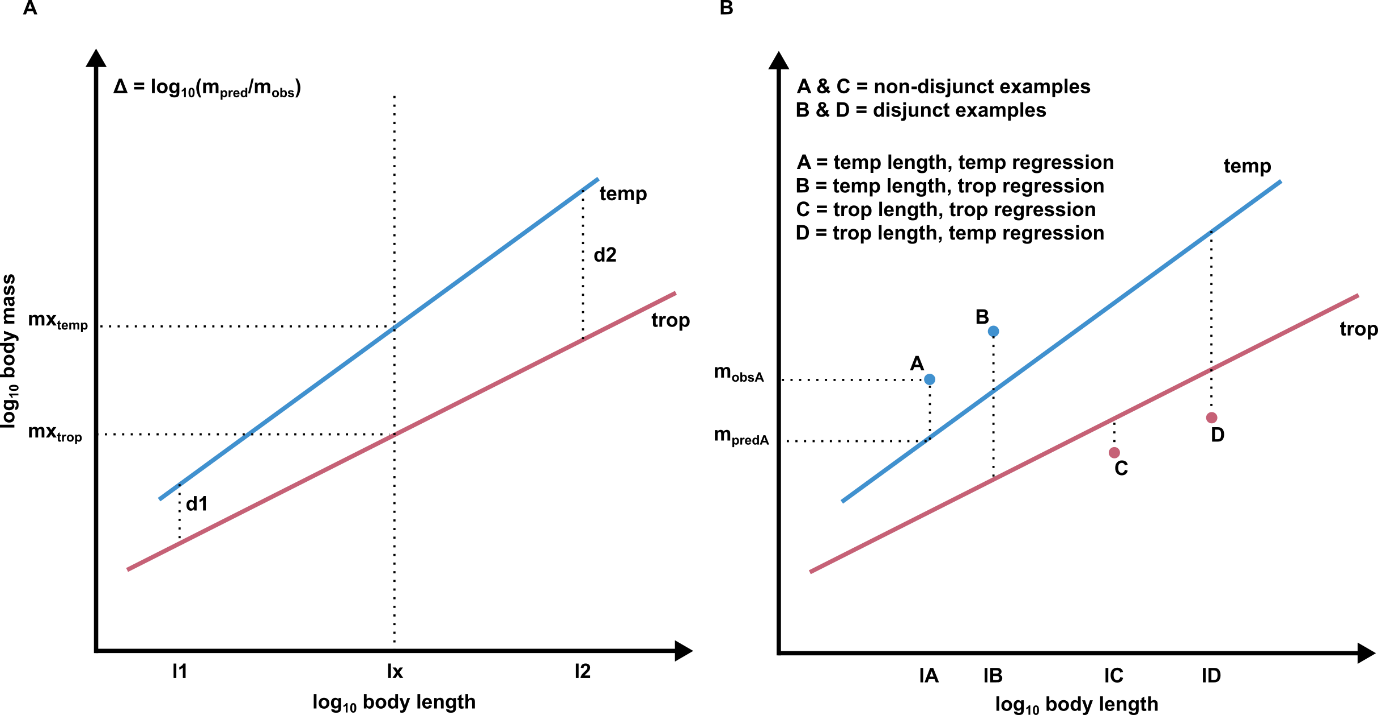
*

Figure S1: Relationship between body length and body mass for all temperate (blue lines) and tropical (red lines) arthropods in our study. Panel A shows that, at a given body length lx, temperate animals in our study were on average heavier than tropical animals. Furthermore, it shows differences in slope (higher for temperate animals) resulting in increasing differences between temperate and tropical body mass along the body-length gradient (at l1, differences between temperate and tropical body mass d1 is smaller than at the higher body length l2). Vertical dotted lines in panel B illustrate differences between observed body masses (example points) and predicted body masses (regression lines) calculated using temperate and tropical disjunct and non-disjunct regression parameters for given body lengths of four example data points (A and B temperate arthropods, C and D tropical arthropods). The vertical distance from data points to disjunct and non-disjunct regression lines (lines representing body-mass prediction for given body lengths using disjunct and non-disjunct regression parameters) illustrates why for disjunct regression parameters prediction accuracy presented in Fig. 2 is higher than for non-disjunct parameters.

In our study, temperate body masses were on average higher than tropical masses at a given body length (Fig. S1 A). The temperate regression for model LR has slope 2.191, while the tropical regression has slope 2.159 (Table 3). This also means that, on average, the difference between temperate and tropical body masses for a given body length increases with body length (d1 and d2). The near-zero discrepancy of non-disjunct predictions is a result of dividing e.g. predicted body mass for temperate organisms by the observed body mass. As these observed body masses were used to obtain the temperate regression parameters (presented in Table 3) for calculating the predicted values, the resulting ratios of m_pred_ and m_obs_ will be near 1 (small deviations from 1 caused by the residuals, the vertical distance between data points and regression lines, see m_obs_A and m_pred_A in Fig. S1 B), resulting in a prediction discrepancy close to log_10_(1)=0. When building this ratio for observed body masses and body masses predicted by geographically disjunct regression parameters (examples B and D in Fig. S1 B), these ratios will more strongly differ from 1 and thus prediction discrepancy will also be higher.

Symmetrical patterns in Fig. 2 panel a/b and c/d are caused by the fact that we employ the temperate and tropical data that was used to obtain the regression parameters in the first place for testing the prediction discrepancy. As can be seen in Fig. S1, our datasets of temperate and tropical animals give rise to a difference in slope and higher average body masses for temperate than tropical animals (for a given body length). Consequently, using this temperate regression for these tropical body masses will result in the same magnitude of over-estimation as using the tropical regressions on temperate data results in an under-estimation. The pattern is slightly more complex for the LWR model simply because this also included body width, but the reason for the symmetry is the same as in the LR model. While this symmetrical pattern is indeed caused by using the same dataset for obtaining the results and testing prediction discrepancy, it does not indicate a methodological issue.

We do not suggest that other studies using geographically disjunct regression parameters will be confronted with exactly these levels of body mass over- or underestimation. All we intend to demonstrate by testing this prediction discrepancy and showing it in Fig. 2 is that using disjunct regression parameters can result in over- or underestimation of body masses and give an example of the extent of prediction discrepancy using our own data. The presented level of prediction discrepancy is specific to our dataset and will vary with the dataset and regression used.
