# Supplementary material for: Applying generalized allometric regressions to predict live body mass of tropical and temperate arthropods
Source: Ecol Evol. 2018 Dec 6;8(24):12737–49. doi: 10.1002/ece3.4702 (PMC6308897; doi:10.1002/ece3.4702)
Supplement: Supplementary file 2 [file ECE3-8-12737-s002.docx]

Supporting Information

Applying generalised allometric regressions to predict live body mass of tropical and temperate arthropods

Esra H. Sohlström^1,2*,^ Lucas Marian^3^, Andrew. D. Barnes^1,3,4,5^, Noor F. Haneda^6^, Stefan Scheu^3^, Björn C. Rall^1,2^, Ulrich Brose^1,2^, Malte Jochum^1,3,4,7^

*Corresponding author: Esra H. Sohlström ([esra.sohlstroem@idiv.de](mailto:esra.sohlstroem@idiv.de))

^1^German Centre for Integrative Biodiversity Research (iDiv) Halle-Jena-Leipzig, Deutscher Platz 5e, 04103 Leipzig, Germany Institute of Biodiversity, Friedrich

^2^Schiller University Jena, Dornburger-Str. 159, 07743 Jena, Germany

^3^J. F. Blumenbach Institute for Zoology & Anthropology, University of Goettingen, Untere Karspüle 2, 37073 Goettingen, Germany

^4^Leipzig University, Institute of Biology, Johannisallee 21, 04103 Leipzig, Germany

^5^School of Science, University of Waikato, Private Bag 3105, Hamilton 3204, New Zealand

^6^Department of Silviculture, Faculty of Forestry, Bogor Agricultural University, Darmaga Campus, Bogor, Indonesia

^7^Institute of Plant Sciences, University of Bern, Altenbergrain 21, 3013 Bern, Switzerland

## Introduction

Here, we will provide straight-forward examples of how to get from individual-level data on body length (mm), body width (mm), taxonomic group and geographic region (temperate or tropical) to individual fresh body mass (mg) using the eight different models from the analysis and data presented in the main text.

We will use the original data to calculate body masses with the eight models. For every model, we will thus subset the overall data set as if we just had a reduced level of information (e.g., data on just length of tropical spiders).

## Step 1: General setup and loading data

First, set the working directory and load the data file.

rm(list=ls())

# Set to your local folder
setwd("...")

# Read the individual-level data
idata <- read.csv("Sohlstroem_individual_data.csv", sep =",")

# See what we got
str(idata)

##

## Step 2: Predict body mass based on eight different models

### Model 1 (LWTR)

This model calculates body mass based on body length (L), body width (W), taxonomic group (T) and geographic region (R) based on the following formula:

$$Bodymass={10}^{intercept_{TR}+slope_{LTR}*log10\left( L \right)+slope_{WTR}*log10\left( W \right)}$$

To simulate a dataset suitable for using this model, we constrain our original individual-level dataset to tropical spiders with length (mm) and width (mm) measured. Subsequently, we calculate fresh body mass (mg) using model LWTR and parameters derived from Table 3 in the main text.

# Subset to tropical spiders:
LWTRdata <- idata[idata$zone=="tropic" & idata$order=="araneae",
 c("zone","length","max_width","order")]

# Calculate fresh body mass:
Bodymass.LWTR = 10^(-0.464+1.539*log10(LWTRdata$length)+1.448*log10(LWTRdata$max_width))

###

### Model 2: (LWT)

This model calculates body mass based on body length (L), body width (W) and taxonomic group (T) based on the following formula:

$$Bodymass={10}^{intercept_{T}+slope*log10\left( L \right)+slope_{WT}*log10\left( W \right)}$$

To simulate a dataset suitable for using this model, we constrain our original individual-level dataset to temperate and tropical spiders with length (mm) and width (mm) measured. Subsequently, we calculate fresh body mass (mg) using model LWT and parameters derived from Table 3 in the main text.

# Subset to temperate and tropical (all) spiders:
LWTdata <- idata[idata$order=="araneae",
 c("zone","length","max_width","order")]

# Calculate fresh body mass:
Bodymass.LWT = 10^(-0.410+1.486*log10(LWTdata$length)+1.492*log10(LWTdata$max_width))

###

### Model 3: (LWR)

This model calculates body mass based on body length (L), body width (W) and geographic region (R) based on the following formula:

$$Bodymass={10}^{intercept_{R}+slope_{LR}*log10\left( L \right)+slope_{WR}*log10\left( W \right)}$$

To simulate a dataset suitable for using this model, we constrain our original individual-level dataset to various tropical arthropods with length (mm) and width (mm) measured. Subsequently, we calculate fresh body mass (mg) using model LWR and parameters derived from Table 3 in the main text.

# Subset to tropical arthropods:
LWRdata <- idata[idata$zone=="tropic",c("zone","length","max_width","order")]

# Calculate fresh body mass:
Bodymass.LWR = 10^(-0.371+1.087*log10(LWRdata$length)+1.647*log10(LWRdata$max_width))

###

### Model 4: (LW)

This model calculates body mass based on just body length (L) and body width (W) based on the following formula:

$$Bodymass={10}^{intercept+slope_{L}*log10\left( L \right)+slope_{W}*log10\left( W \right)}$$

To simulate a dataset suitable for using this model, we constrain our original individual-level dataset to various arthropods from temperate and tropical regions with length (mm) and width (mm) measured. Subsequently, we calculate fresh body mass (mg) using model LW and parameters derived from Table 3 in the main text.

# Subset to all temperate and tropical arthropods:
LWdata <- idata[,c("zone","length","max_width","order")]

# Calculate fresh body mass:
Bodymass.LW = 10^(-0.340+1.070*log10(LWdata$length)+1.634*log10(LWdata$max_width))

###

### Model 5: (LTR)

This model calculates body mass based on body length (L), taxonomic group (T) and geographic region (R) based on the following formula:

$$Bodymass={10}^{intercept_{TR}+slope_{LTR}*log10\left( L \right)}$$

To simulate a dataset suitable for using this model, we constrain our original individual-level dataset to tropical spiders with just length (mm) measured. Subsequently, we calculate fresh body mass (mg) using model LTR and parameters derived from Table 3 in the main text.

# Subset to tropical spiders:
LTRdata <- idata[idata$zone=="tropic" & idata$order=="araneae",c("zone","length","order")]

# Calculate fresh body mass:
Bodymass.LTR = 10^(-0.862+2.611*log10(LTRdata$length))

###

### Model 6: (LT)

This model calculates body mass based on just body length (L) and taxonomic group (T) based on the following formula:

$$Bodymass={10}^{intercept_{T}+slope*log10\left( L \right)}$$

To simulate a dataset suitable for using this model, we constrain our original individual-level dataset to spiders from temperate and tropical regions with just length (mm) measured. Subsequently, we calculate fresh body mass (mg) using model LT and parameters derived from Table 3 in the main text.

# Subset to temperate and tropical spiders:
LTdata <- idata[idata$order=="araneae",c("zone","length","order")]

# Calculate fresh body mass:
Bodymass.LT = 10^(-0.830+2.637*log10(LTdata$length))

###

### Model 7: (LR)

This model calculates body mass based on just body length (L) and geographic region (R) based on the following formula:

$$Bodymass={10}^{intercept_{R}+slope_{LR}*log10\left( L \right)}$$

To simulate a dataset suitable for using this model, we constrain our original individual-level dataset to various tropical arthropods with just length (mm) measured. Subsequently, we calculate fresh body mass (mg) using model LR and parameters derived from Table 3 in the main text.

# Subset to all tropical arthropods:
LRdata <- idata[idata$zone=="tropic",c("zone","length","order")]

# Calculate fresh body mass:
Bodymass.LR = 10^(-0.826+2.159*log10(LRdata$length))

###

### Model 8: (L)

This model calculates body mass based on just body length (L) based on the following formula:

$$Bodymass={10}^{intercept+slope_{L}*log10\left( L \right)}$$

To simulate a dataset suitable for using this model, we constrain our original individual-level dataset to various arthropods from temperate and tropical regions with just length (mm) measured. Subsequently, we calculate fresh body mass (mg) using model L and parameters derived from Table 3 in the main text.

# Subset to all temperate and tropical arthropods:
Ldata <- idata[,c("zone","length","order")]

# Calculate fresh body mass:
Bodymass.L = 10^(-0.792+2.181*log10(Ldata$length))
